# Crystal Structures of Human CaMKIα Reveal Insights into the Regulation Mechanism of CaMKI

**DOI:** 10.1371/journal.pone.0044828

**Published:** 2012-09-20

**Authors:** Manwu Zha, Chen Zhong, Ying Ou, Li Han, Jianchuan Wang, Jianping Ding

**Affiliations:** State Key Laboratory of Molecular Biology, Institute of Biochemistry and Cell Biology, Shanghai Institutes for Biological Sciences, Chinese Academy of Sciences, Shanghai, China; University of Oulu, Finland

## Abstract

Human calcium/calmodulin-dependent protein kinase I (CaMKI) plays pivotal roles in the nervous system. The activity of human CaMKI is regulated by a regulatory region including an autoinhibitory segment and a CaM-binding segment. We report here four structures of three CaMKIα truncates in apo form and in complexes with ATP. In an apo, autoinhibited structure, the activation segment adopts a unique helical conformation which together with the autoinhibitory segment constrains helices αC and αD in inactive conformations, sequesters Thr177 from being phosphorylated, and occludes the substrate-binding site. In an ATP-bound, inactive structure, the activation segment is largely disordered and the CaM-binding segment protrudes out ready for CaM binding. In an ATP-bound, active structure, the regulatory region is dissociated from the catalytic core and the catalytic site assumes an active conformation. Detailed structural analyses reveal the interplay of the regulatory region, the activation segment, and the nucleotide-binding site in the regulation of CaMKI.

## Introduction

Intracellular calcium is an important secondary messenger, of which the concentration ranges from a basal value of about 50 nM to stimulated levels of 1–10 µM in response to signals such as growth factors and neurotransmitters [1]. One of the key proteins that sense the increased calcium concentration is calmodulin (CaM), which consists of four EF hands [2]. When activated, CaM binds to and stimulates the activities of a family of Ca^2+^/CaM-dependent serine/threonine protein kinases (CaMKs), thereby regulating their functions. The CaMKs regulated by Ca^2+^/CaM include mono-functional kinases myosin light-chain kinase and phosphorylase kinase, and multi-functional enzymes CaMKI, CaMKII, CaMKIV, and CaMK kinase (CaMKK).

CaMKI plays pivotal roles in the nervous system. It is critical for long-term potentiation via activation of ERK [3] and recruitment of synaptic Ca^2+^-permeable AMPARs [4]. It also promotes dendritic arborization [5], neurite outgrowth [6], and formation of spines, synapses and axons in hippocampal neurons [7], [8]. Besides exerting important functions in the nervous system, CaMKI might also be involved in osteoclast differentiation and bone resorption [9]. The kinase recognizes a consensus sequence Hyd-X-Arg-X-X-Ser/Thr-X-X-X-Hyd, in which Hyd is a hydrophobic residue [10], and its substrates include the synaptic vesicle-associated proteins, namely synapsin 1 and 2 [11], the cAMP response element-binding protein (CREB) [12], and the recently identified target glial cell missing 1 (GCM1) [13].

It has been shown that the kinase activity of CaMKI is regulated by a C-terminal regulatory region which contains an autoinhibitory segment (residues 286–307) and an overlapping CaM-binding segment (residues 303–316) [14], [15]. In particular, three truncation forms of CaMKI exhibit distinct characteristics in basal kinase activity and responsiveness to Ca^2+^/CaM: the truncate encompassing residues 1–321 which contains both the autoinhibitory segment and the CaM-binding segment displays properties similar to those of the full-length protein; the truncate encompassing residues 1–314 which contains the autoinhibitory segment has a substantially lower kinase activity than the full-length kinase although it can bind Ca^2+^/CaM; and the truncate encompassing residues 1–293 which excludes the regulatory region is constitutively active and exhibits an activity comparable to that of the full-length kinase [14].

The crystal structure of the apo rat CaMKI320 (residues 1–320) has been determined, leading to the proposal that the regulatory region of CaMKI inhibits the kinase activity by interacting with the N-terminal lobe and hence occluding the nucleotide-binding site and restraining the kinase in an inactive conformation [16]. However, the regulation mechanism of CaMKI remains elusive. Here we report four crystal structures of three truncation variants of human CaMKIα, namely CaMKI320 (residues 1–320), CaMKI315 (residues 1–315), and CaMKI293 (residues 1–293), which correspond to the aforementioned three truncation forms of CaMKI [14], respectively. The structural data reveal new insights into the regulation mechanism of CaMKI.

## Materials and Methods

### Cloning, expression, and purification of CaMKI proteins

The cDNA fragments encoding different human CaMKIα truncates were inserted into the BamHI and SalI restriction sites of the pGEX4T-1 expression plasmid (Novagen) which attaches a GST tag at the N-terminus of the protein. The plasmids were transformed into *E. coli* BL21(DE3) strain (Novagen), and the transformed cells were grown in LB medium at 37°C in the presence of 50 µg/ml ampicillin until OD_600_ reached 0.8 and then induced with 0.5 mM IPTG for 12 hours at 16°C. The cells were harvested and lysed by sonication in a lysis buffer (140 mM NaCl, 2.7 mM KCl, 10 mM Na_2_HPO_4_, and 1.8 mM KH_2_PO_4_, pH 7.3). Protein purification was carried out by affinity chromatography using a GSTrap FF 5 ml column (GE healthcare). Cleavage of the fusion proteins with thrombin protease on column was conducted at 16°C for 24 hours. The elution fractions were further purified by gel filtration using a Superdex 200 16/60 column (GE healthcare). The target proteins were concentrated to about 10 mg/ml with Centricon-10 (Millipore) for structural studies.

### Crystallization and diffraction data collection

Crystallization was performed using the hanging drop vapor diffusion method. The drop consisted of equal volumes of the protein solution and the reservoir solution. The protein solution contained 10 mg/ml CaMKI protein in the lysis buffer alone or supplemented with 20 mM MgCl_2_ and 5 mM ATP. For the apo CaMKI320, the crystals were grown at 18°C and the reservoir solution contained 2.0 M (NH_4_)_2_SO_4_ and 100 mM bicine, pH 8.0. For the CaMKI320-ATP complex, the crystals were grown at 18°C and the reservoir solution contained 0.5 M (NH_4_)_2_SO_4_, 0.8 M Li_2_SO_4_, and 100 mM tri-sodium citrate, pH 5.6. For the CaMKI315-ATP complex, the crystals were grown at 4°C and the reservoir solution contained 1.5 M (NH_4_)_2_SO_4_, 3% dioxane, and 100 mM MES, pH 6.0. For the CaMKI293-ATP complex, the crystals were grown at 4°C and the reservoir solution contained 25% polyethylene glycol 8,000, 200 mM NH_4_Ac, and 100 mM (CH_3_)_2_AsO_2_Na, pH 6.0. The diffraction data of the CaMKI293-ATP complex were collected at beamline BL-6A of Photon Factory, Japan; those of the CaMKI320-ATP complex at beamline 1W2B of Beijing Synchrotron Radiation Facility, China; and those of the apo CaMKI320 and the CaMKI315-ATP complex at beamline 17U of Shanghai Synchrotron Radiation Facility, China. The data were processed, integrated, and scaled together using the HKL2000 suite [17]. For apo CaMKI320, the crystal used for data collection had a little bit ice, and hence during data processing the data in the resolution shell of 3.5–3.8 Å were largely excluded which yielded a relatively low overall completeness of the whole dataset. The statistics of the diffraction data are summarized in **Table 1**.

**Table 1:** Data collection and refinement statistics.

	apo CaMKI320	CaMKI320-ATP	CaMKI315-ATP	CaMKI293-ATP
Data collection				
Space group	*C*2	*P*6_3_	*P*6_3_	*P*4_1_2_1_2
*a* (Å)	91.1	83.1	83.4	65.7
*b* (Å)	67.7	83.1	83.4	65.7
*c* (Å)	56.1	153.0	153.4	139.9
α, β, γ (°)	90, 99.8, 90	90, 90, 120	90, 90, 120	90, 90, 90
Resolution (Å)	50.0-2.60	50.0-2.40	50.0-2.20	50.0-2.70
	(2.69-2.60)*	(2.49-2.40)	(2.28-2.20)	(2.80-2.70)
*R*_merge_ (%)	12.1 (29.3)	6.3 (49.8)	7.2 (43.7)	5.2 (42.5)
<*I*/σ*I*)>	11.4 (3.9)	19.1 (2.6)	22.4 (3.6)	33.6 (4.9)
Completeness (%)	88.6 (100.0)	99.9 (100.0)	99.1 (99.4)	99.4 (99.8)
Redundancy	4.1 (4.4)	4.7 (4.7)	5.5 (5.5)	7.5 (6.5)
**Refinement**				
No. reflections	8,749	22,205	28,786	8,457
*R*_work_/*R*_free_ (%)	25.1/29.9	21.1/24.9	19.8/24.8	22.1/24.6
No. atoms				
Protein	2,227	4,410	4,189	2,003
Ligand		2	2	1
Water	58	136	135	79
RMSD				
Bond lengths (Å)	0.005	0.006	0.009	0.016
Bond angles (°)	0.9	1.2	1.3	1.4
Luzatti coordinate error (Å)	0.38	0.42	0.32	0.39
Ramachandran plot (%)				
Most favored	89.3	90.4	91.6	90.9
Allowed	10.3	9.2	7.8	8.6
Generously allowed	0.4	0.4	0.6	0.5

* Values in parentheses are for highest-resolution shell.

### Structure determination and refinement

The structure of the CaMKI293-ATP complex was solved with the molecular replacement method implemented in program CNS [18] using the apo rat CaMKI320 structure (PDB code 1A06) as the search model. The other structures of CaMKI were solved with the molecular replacement method using the structure of the CaMKI293-ATP complex as the search model. The model building was performed using Coot [19], and the structure refinement using programs CNS [18] and REFMAC5 [20]. The statistics of the structure refinement and the structure models are also summarized in **Table 1**.

## Results

### Overall structure

**Figure 1:**
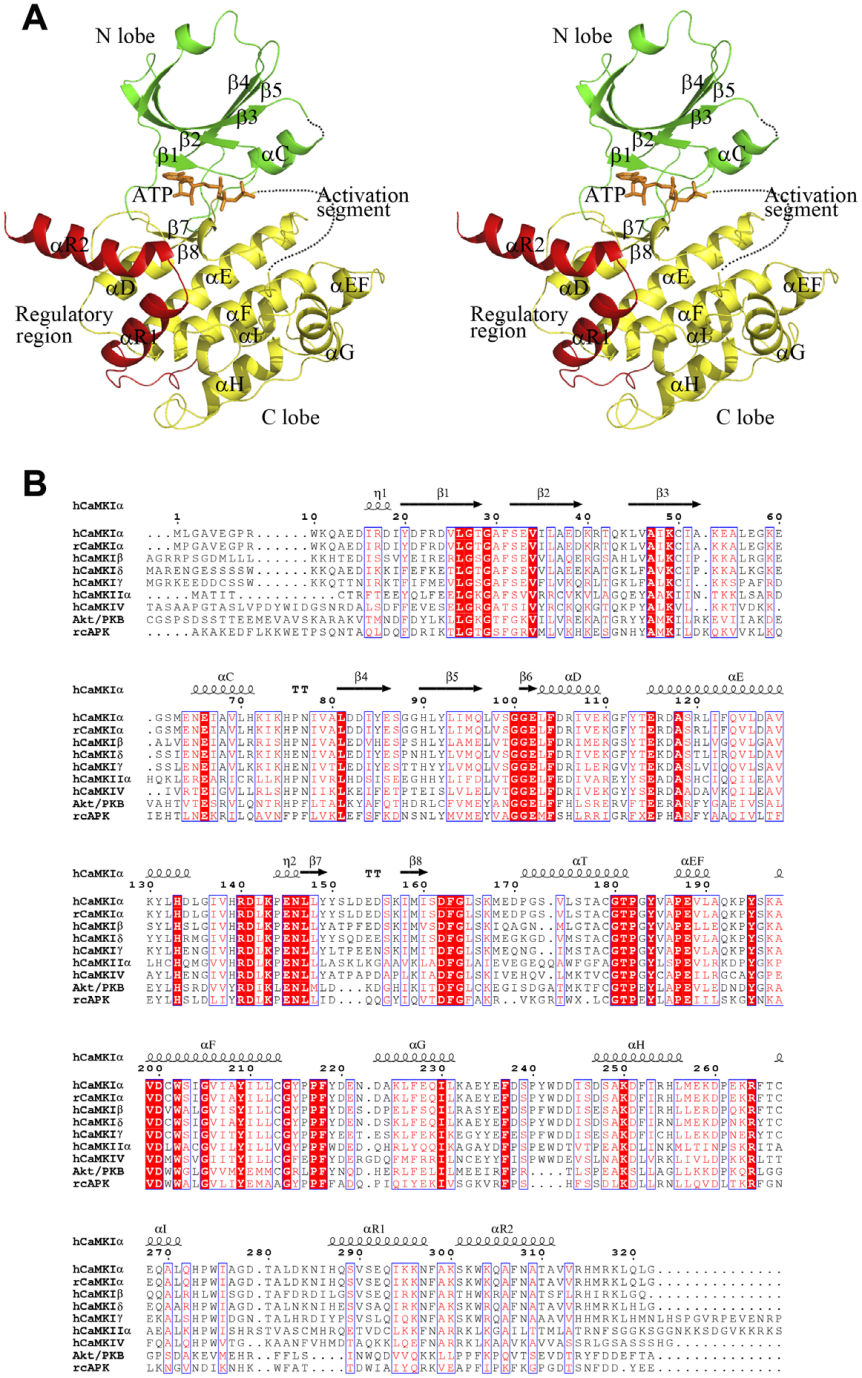
Structure of CaMKI. **A** A stereoview of the overall structure of CaMKI320-ATP. The various CaMKI truncation forms have similar overall structure and the CaMKI320-ATP complex is selected as a representative. CaMKI consists of a catalytic core containing the N lobe (green), the C lobe (yellow) and a regulatory region (red). The bound ATP is shown with a ball-and-stick model (orange). The activation segment is primarily disordered and denoted with a dashed curve in orange. **B** Sequence alignment of human CaMKI (isoforms α, β, δ, and γ), rat CaMKIα, human CaMKIIα, human CaMKIV, human Akt/PKB, and rat cAPK. The highly conserved residues are indicated with closed red boxes, and the conserved residues with open red boxes. The secondary structure elements of human CaMKIα are indicated on the top of the alignment.

To explore the molecular mechanism underlying the regulation of CaMKI activity, we obtained the crystal structures of CaMKI320, CaMKI315, and CaMKI293 which diverge in the regulatory region. The structures of CaMKI320 were determined in apo form and in complex with ATP, CaMKI315 in complex with ATP, and CaMKI293 in complex with ATP, respectively (Table 1). These structures are similar in the overall conformation with root mean square deviations of 0.6–1.6 Å for 244 Cα atoms. The structure of CaMKI320-ATP is shown in Figure 1A as a representative, and the nomenclature of the secondary structure elements are named after that of cAPK [21] (Figure 1B). The catalytic core of CaMKI takes a canonical bilobate architecture, with the N-terminal lobe (N lobe) consisting of five antiparallel β-strands and a prominent helix named helix αC and the C-terminal lobe (C lobe) comprised primarily of α-helices. The active site is formed at the interface of the two lobes where ATP is bound. The C-terminal region (residues 276–315) containing the autoinhibitory and CaM-binding sequences assumes a helix-loop-helix structure. Although the overall conformation is similar, detailed analyses of these structures reveal their distinctive features in the activation segment, the nucleotide-binding site, and the regulatory region, which together provide new insights into the regulation mechanism of CaMKI.

### A unusual helical conformation of the active segment

**Figure 2:**
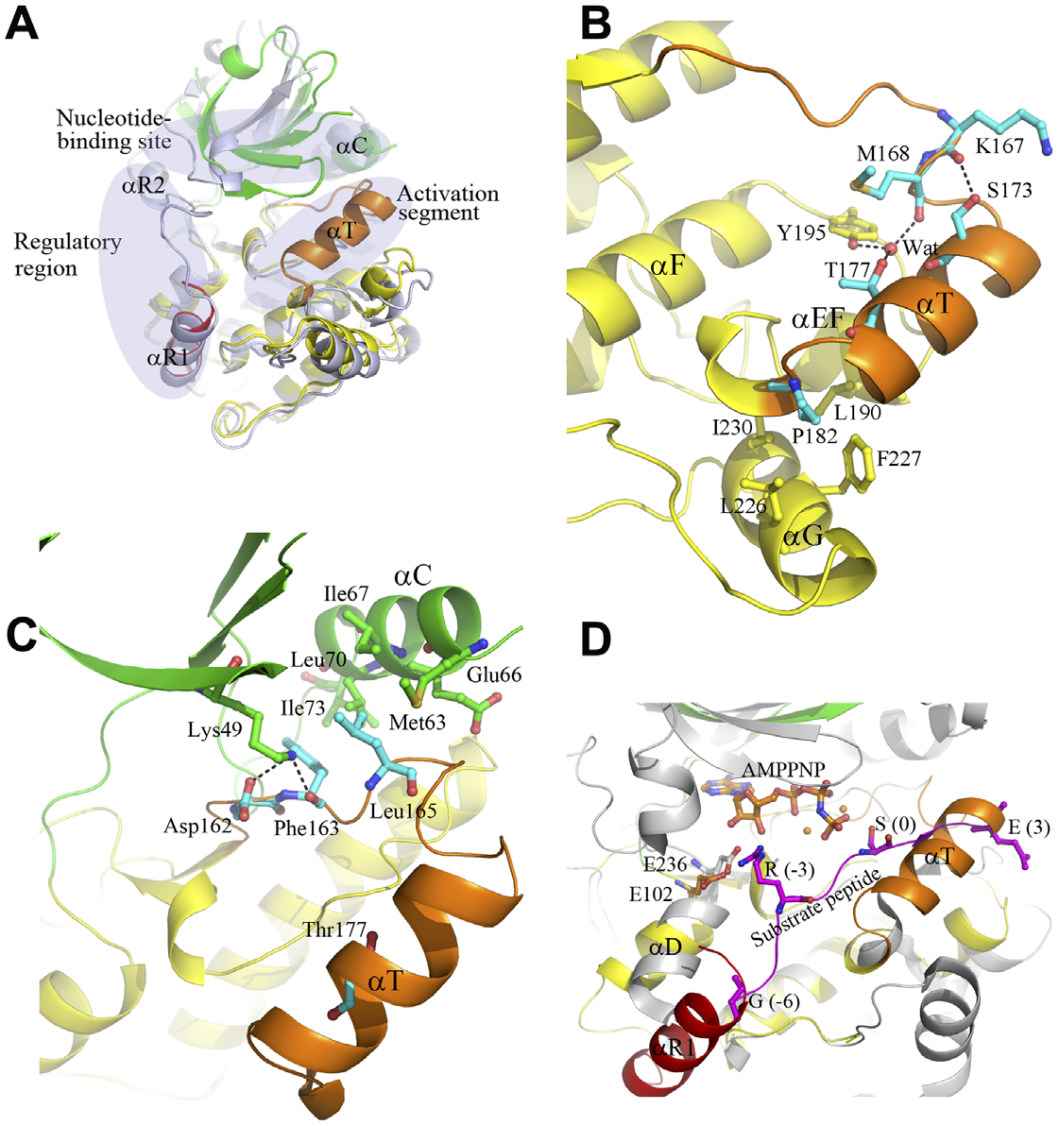
A distinctive autoinhibited state of CaMKI. **A** Comparison of the apo human CaMKI320 and the apo rat CaMKI320 (PDB code 1A06) [16]. The apo human CaMKI320 is colored as in Figure 1A. The apo rat CaMKI320 is colored in light blue. Substantial conformational differences are observed between the two structures in the regulatory region, the activation segment, and the nucleotide-binding site which are indicated with ovals. In particular, the C-terminal part of the activation segment encompassing residues Pro171 to Thr181 assumes a rare helical conformation (helix αT). **B** Hydrophilic and hydrophobic interactions of helixαT with the surrounding residues in the apo CaMKI320. The involved residues are shown with ball-and-stick models, and the hydrogen-bonding interactions are indicated with dashed lines. **C** Interactions of the N-terminal part of the activation segment with the N lobe in the apo CaMKI320. The N-terminal part of the activation segment including the DFG motif and the following Leu165 form both hydrophilic and hydrophobic interactions with helix αC and strand β3 of the N lobe. The involved residues and additionally Glu66 on helix αC are shown with ball-and-stick models, and the hydrogen-bonding interactions are indicated with dashed lines. **D** Comparison of the apo CaMKI320 and the Akt/PKB-GSK3β complex in the substrate-binding site. In the Akt/PKB-GSK3β complex (gray), the bound GSK3β peptide is shown with a magenta ribbon, and the bound AMPPNP is shown with a ball-and-stick model in orange. Residues at P(-6), P(-3), P0, and P+3 positions of the GSK3β peptide, Glu236 of Akt/PKB which forms a salt bridge with Arg at P(-3) of the peptide, and the equivalent Glu102 in the apo CaMKI320 are shown with ball-and-stick models.

Surprisingly, structural comparison shows that the apo human CaMKI320 structure obtained in this study is substantially different from the apo rat CaMKI320 structure [16], particularly in the activation segment, the nucleotide-binding site, and the regulatory region (Figure 2A). In the rat CaMKI320, the activation segment is disordered and the phosphate-binding loop (P-loop, the β1-β2 hairpin) [22] takes an unusual conformation with residues 29–34 forming a distorted 3_10_ helix. In addition, the regulatory region runs across the catalytic core with its C-terminus interacting with the N lobe, leading to an open conformation of the overall structure. It was proposed that the aberrant conformation of the P-loop and the obstruction of the nucleotide-binding site by helix αR2 and the αR1-αR2 loop of the regulatory region contribute to autoinhibition of the enzyme [16]. In contrast, in our CaMKI320, the P-loop takes a canonical conformation, the C-terminus of the regulatory region (residues 300–320) is undetected, and the nucleotide-binding site is amenable to nucleotide binding. More intriguingly, the C-terminal part of the activation segment encompassing residues Pro171 to Thr181 assumes a unique helical conformation (designated as helix αT) (Figure 2A) with well-defined electron density (an average B factor of 39.8 Å^2^) and the DFG motif at the N-terminal part of the activation segment takes an “in” conformation. The rare helical conformation of αT is stabilized by the hydrophilic and hydrophobic interactions within the activation segment and with the surrounding secondary structure elements including helices αEF and αG and the αEF-αF loop (Figure 2B). Specifically, the hydroxyl of Thr177 (the phosphorylation site of the activation segment) forms hydrogen-bonding interactions indirectly with the hydroxyl of Tyr195 (a strictly conserved residue on the αEF-αF loop) and the main-chain carbonyl of Met168 via a water molecule, and the hydroxyl of Ser173 forms a hydrogen bond with the main-chain carbonyl of Lys167. In addition, Pro182 of the activation segment C-terminal to helix αT forms hydrophobic interactions with Leu190 of helix αEF and Leu226, Phe227, and Ile230 of helix αG, further stabilizing the specific conformation of the activation segment (Figure 2B).

On the other hand, helix αT also forms a part of the interface of two two-fold symmetry-related molecules, and we performed crystal packing analysis to examine whether the conformation of helix αT is constrained by the crystal lattice. In the apo CaMKI320 belonging to space group C2, two-symmetry related molecules are juxtaposed head to tail, and the interface is formed mainly between helix αT of one molecule and helices αG and αEF of the other (Figure S1A). The interface is stabilized mainly by hydrophobic interactions. Particularly, the side chains of Pro171, Leu175, and Cys179 of helix αT of one molecule make hydrophobic contacts with the side chains of Phe227 of helix αG and Leu190 of helix αEF of the other, forming a hydrophobic cluster (Figure S1A). Interestingly, the unique conformation of helixαT captured in the apo CaMKI320 is also observed in the structure of CaMKI293 in complex with an inhibitor which is crystallized in a different space group (*I*222), and in that structure the interface is formed by similar structure elements of two two-fold symmetry-related molecules but involves different interacting residues (unpublished data). In addition, in the CaMKI320-ATP and CaMKI315-ATP structures which are crystallized in another space group (*P*6_3_), two pseudo two-fold symmetry-related molecules are juxtaposed head to tail in a similar way as in the apo CaMKI320. Consistently, the interface is comparable to that in the apo CaMKI320 and particularly, helices αG and αEF occupy similar positions as those in the apo CaMKI320 structure (Figure S1B). However, helix αT is largely disordered in one molecule and completely disordered in the other, although the hydrophobic cluster at the interface is partially maintained (Figure S1B). In contrast, in the CaMKI293-ATP structure which is crystallized in space group *I*222, the C-terminal part of the activation segment is completely disordered, and the two symmetry-related molecules are juxtaposed head to head. Correspondingly, the interface is substantially different from those in the CaMKI320, CaMKI320-ATP, and CaMKI315-ATP structures. Taken together, these results indicate that the activation segment has a high flexibility and the crystal packing interactions do not contribute to the formation of helix αT. Thus, the apo CaMKI320 structure with a helix αT-containing activation segment should represent a biologically relevant state of CaMKI.

### A distinctive autoinhibited state of CaMKI

To explore the biological relevance of the apo CaMKI320, we compared it with the structure of the Akt/PKB-GSK3β complex [23]. Akt/PKB belongs to the AGC family which is the most closely related to the CaMK family [24], and the Akt/PKB-GSK3β complex represents an active state of the enzyme [23]. The comparison reveals substantial conformational differences especially at helix αC and the activation segment. It was shown previously that the conformation of helix αC is critical for the maintenance of the nucleotide-binding site and the coordination of the conformational changes at the catalytic site [23], [25]. In the active Akt/PKB-GSK3β complex, helix αC is coupled to the nucleotide-binding site and the activation segment via a salt-bridging interaction between two conserved residues in the N lobe, an invariant Glu (Glu66 in CaMKI) of helix αC and a highly conserved Lys (Lys49 in CaMKI) of strand β3, which is a hallmark of the active kinase [25]. In the apo CaMKI320, Lys49 interacts with Asp162 and Phe163 of the DFG motif of the activation segment (Figure 2C). Concurrently, helix αC is rotated outward and Glu66 points its side chain away from the catalytic site, making it impossible to interact with Lys49. This inactive conformation of helix αC is maintained largely by the hydrophobic contacts between Phe163 and Leu165 of the activation segment and Met63, Ile67, Leu70, and Ile73 of the hydrophobic surface of helix αC (Figure 2C).

In many kinases, phosphorylation of the activation segment can enhance the kinase activity [25]. In Akt/PKB-GSK3β, Thr309 of the activation segment is phosphorylated and the activation segment takes an extended loop conformation which is stabilized by electrostatic interactions via the phosphate group of phosphorylated Thr309, leaving the catalytic site open for substrate binding. Whereas in CaMKI320, the activation segment including helix αT and the β8-αT loop occupies part of the catalytic site. Although unphosphorylated, the side chain of Thr177 (equivalent to Thr309 of Akt/PKB) is oriented inwards and forms interactions with the surrounding residues to stabilize the specific conformation of the activation segment, shielding it from surface exposure and hence from CaMKK phosphorylation (Figure 2A). It was reported that Thr177 of the full-length CaMKI cannot be phosphorylated in the absence of Ca^2+^/CaM, and that an inactive form of CaMKI (CaMKI306) which contains the autoinhibitory segment but not the CaM-binding segment cannot be phosphorylated either in the presence or absence of Ca^2+^/CaM [26]. The previously reported apo rat CaMKI320 structure could not explain the unresponsiveness of the inactive CaMKI306 to CaMKK as the activation segment is completely disordered [16]. In contrast, the unusual helical conformation of the activation segment observed in our apo CaMKI320 structure and the resulted sequestration of Thr177 provide a reasonable explanation for the biochemical data [26].

The consensus sequence of the substrates of CaMKI (Hyd-X-Arg-X-X-Ser/Thr-X-X-X-Hyd) is related to that of Akt/PKB (Arg-X-X-Arg-X-X-Ser/Thr-Hyd), both of which contain an Arg at the P(-3) position. The conservation of Akt/PKB and CaMKI and their substrates suggest that CaMKI might bind its substrate in a way similar to that of Akt/PKB with the GSK3β peptide. In the Akt/PKB-GSK3β complex, Glu236 of the hinge region (the β5-αD loop) forms a salt bridge with Arg at P(-3) of the substrate peptide [9]. In the apo CaMKI320, the equivalent Glu102 occupies the same position as Glu236 of Akt/PKB, suggesting that the interaction between Glu102 and Arg at P(-3) of a CaMKI substrate might be conserved (Figure 2D). On the other hand, there are obvious steric conflicts between the residues at P+1 to P+3 of the GSK3β peptide and the N-terminus of helix αT and between the residues at P(-5) and P(-6) of the GSK3β peptide and the C-terminus of helix αR1 of the autoinhibitory segment (see details later) (Figure 2D), suggesting that helix αT and helix αR1 in the apo CaMKI320 might interfere with substrate binding due to potential steric blockage of the substrate-binding site. These results together suggest that the unique helix αT of the activation segment together with the autoinhibitory segment observed in the apo CaMKI320 restrains helix αC in an inactive conformation, sequesters Thr177 of the activation segment from being phosphorylated, and blocks the substrate-binding site, leading to the autoinhibition of CaMKI, and thus the apo CaMKI320 represents a distinctive autoinhibited state of CaMKI.

### Two conformational states of ATP-bound CaMKI

**Figure 3:**
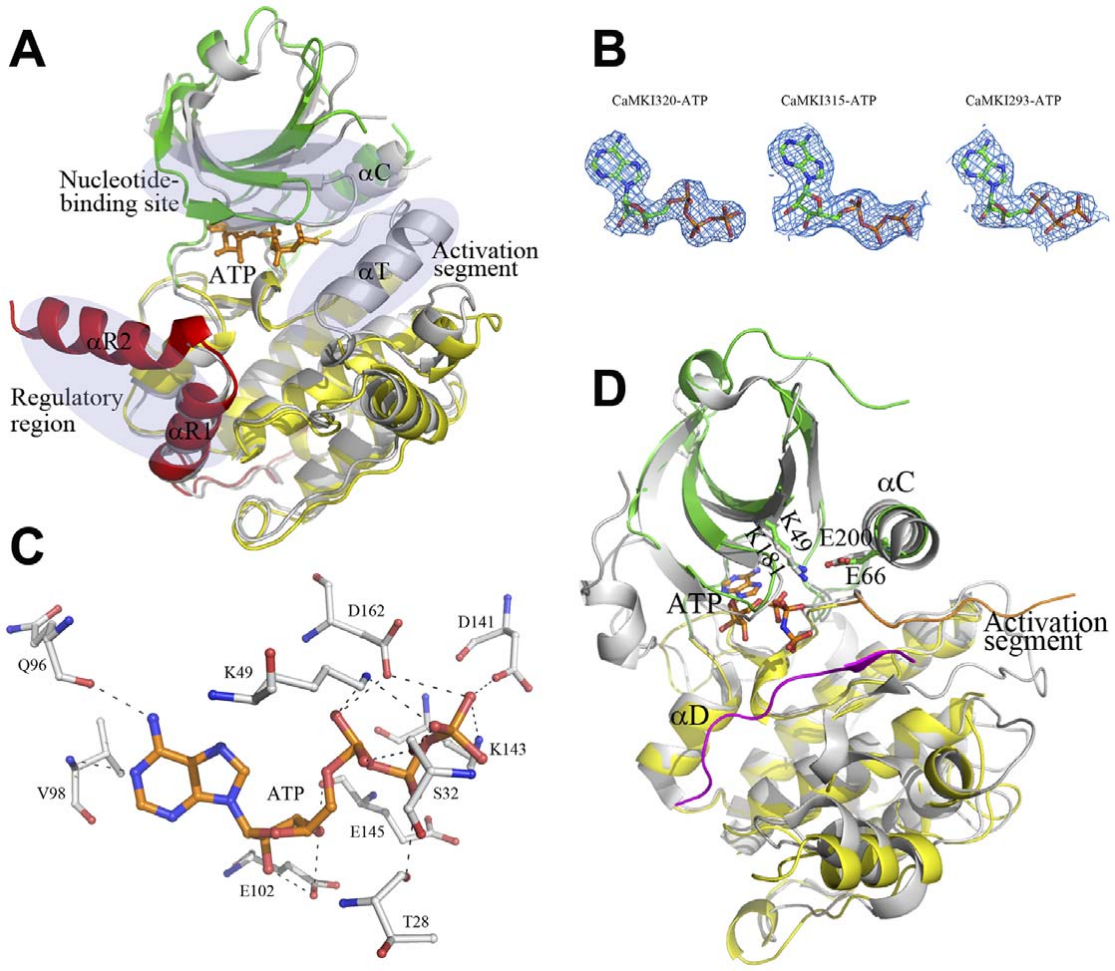
Structures of the ATP-bound CaMKI. **A** Comparison of the CaMKI320-ATP complex with the apo CaMKI320. The CaMKI320-ATP complex is colored as in Figure 1A, and the apo CaMKI320 is colored in gray. The three regions where the CaMKI320-ATP complex shows substantial conformational differences from the apo CaMKI are indicated with ovals. **B** The 2*F*o-*F*c maps of the bound ATP molecules (contoured at 1σ level) in the CaMKI320-ATP, CaMKI315-ATP, and CaMKI293-ATP structures. **C** Hydrophilic interactions of ATP with the surrounding residues in CaMKI320-ATP. ATP and the interacting residues are shown with ball-and-stick models, and the hydrogen-bonding interactions are indicated with dashed lines. **D** Comparison of the CaMKI293-ATP complex and the Akt/PKB-GSK3β complex at the catalytic site. The color coding is the same as in Figures 1A and 2D. The bound ATP is shown with a ball-and-stick model. Glu66 on helix αC and Lys49 on strand β3 in CaMKI293-ATP and their equivalents (Glu200 and Lys181) in Akt/PKB-GSK3β are shown with ball-and-stick models.

As described above, unlike the rat apo CaMKI320, in the apo CaMKI320 the nucleotide-binding site is not blocked by the regulatory region and thus is amenable to nucleotide binding. Therefore, we co-crystallized CaMKI320 with ATP and successfully obtained the complex structure (Figure 3A), in which ATP is observed with evident electron density; however, no Mg^2+^ was visible despite the presence of 20 mM MgCl_2_ in the protein solution (Figure 3B). Upon ATP binding, CaMKI320 undergoes significant conformational changes characterized by a different N lobe orientation, the disordering of helix αT of the activation segment, and the formation of helix αR2 of the CaM-binding segment compared with the apo CaMKI320 (Figure 3A). In particular, the residues on strand β1 of the P-loop exhibit positional displacements of about 5–7 Å, and ATP bound at the nucleotide-binding site interacts with the surrounding residues via a network of hydrogen bonds (Figure 3C). Specifically, the N1 and N6 atoms of the adenosine form hydrogen bonds with the main-chain amide of Val98 and the main-chain carbonyl of Gln96, respectively. The 2′- and 3′-hydroxyls of the ribose moiety form hydrogen bonds with the side-chain carboxyl of Glu102, and additionally, the 3′-hydroxyl forms a hydrogen bond with the main-chain carbonyl of Glu145. The α- phosphate forms hydrophilic interactions with the side-chain amine of Lys49 of strand β3, the side-chain carboxyl of Asp162 of the DFG motif, and the side-chain hydroxyl of Ser32 of the P-loop. The β-phosphate forms a hydrogen bond with the main-chain carbonyl of Thr28 of the P-loop. The γ-phosphate interacts with the side-chain carboxyl of Asp141 and the side-chain amine of Lys143 of the catalytic loop (the αE-β7 loop) and the side-chain carboxyl of Asp162. The CaMKI320-ATP complex displays structural features of an inactive conformation. Compared with the apo CaMKI320, although the structure elements forming the nucleotide-binding site show significant positional displacements up to 7 Å and helix αC is rotated towards strands β3-β5, Glu66 and Lys49 does not form a salt bridge which is critical for the maintenance of the active kinase.

Additionally, we also obtained the structures of CaMKI315 and CaMKI293 in complexes with ATP. CaMKI315 containing the autoinhibition segment cannot be activated by CaM as the CaM-binding segment is incomplete [14]. The CaMKI315-ATP structure is very similar to the CaMKI320-ATP structure and also adopts an inactive conformation. The bound ATP shows evident electron density (Figure 3B) and maintains similar interactions with the enzyme as in the CaMKI320-ATP complex; however, the phosphate groups of ATP have a much higher average B factor and no Mg^2+^ was observed in the electron density.

On the other hand, the CaMKI293-ATP structure shows notable differences from the CaMKI320-ATP structure in the overall conformation and at the nucleotide-binding site (Figure 3A). CaMKI293 lacks the autoinhibitory segment and exhibits a constitutive activity comparable to that of the full-length enzyme [14]. Consistently, the CaMKI293-ATP structure resembles the Akt/PKB-GSK3β structure and displays the structural features of an active conformation (Figure 3D). Specifically, helix αC assumes a position similar to that in the active Akt/PKB-GSK3β complex, and concomitantly Glu66 of helix αC adopts the same conformation as that of Glu200 of Akt/PKB and forms a salt bridge with Lys49. The bound ATP maintains similar interactions with the enzyme as in the CaMKI320-ATP and CaMKI315-ATP complexes but the phosphate groups of ATP also have a high average B factor, indicating a high flexibility that probably allows conformational changes to occur readily when the metal ion and/or the substrate bind to the catalytic site.

### Functional roles of the regulatory region

**Figure 4:**
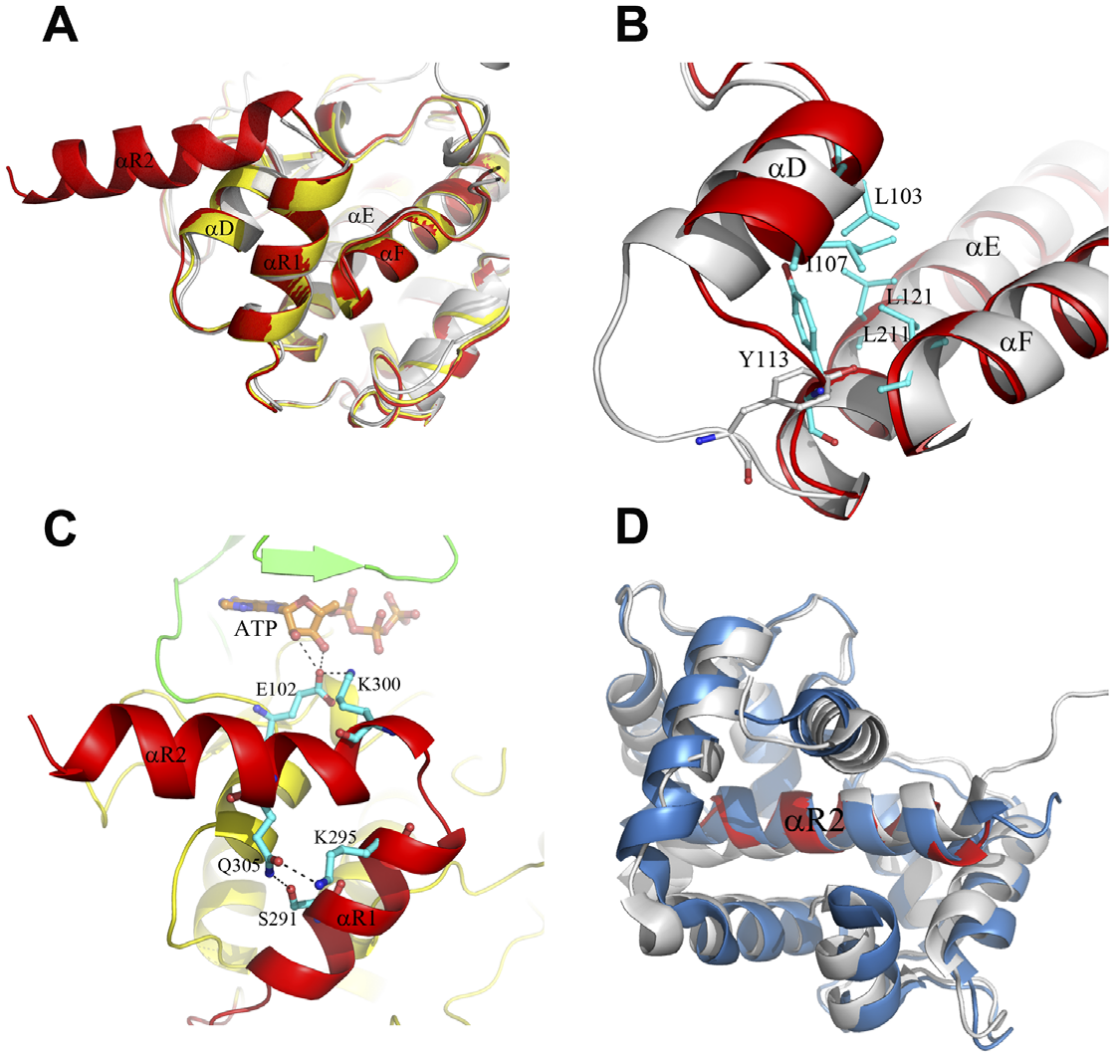
Structures of the regulatory region. **A** Comparison of the regulatory region in the apo CaMKI320 (gray), and the CaMKI320-ATP (red) and CaMKI315-ATP (yellow) complexes. In all three structures, the autoinhibitory segment (helix αR1) interlocks with helices αD and αF. **B** Comparison of the conformations of helix αD and the αD-αE loop in the CaMKI320-ATP (gray) and CaMKI293-ATP (red) complexes. The involved residues are shown with ball-and-stick models and colored in cyan. **C** Structure of the regulatory region in the CaMKI320-ATP complex showing the functional roles of helix αR2. CaMKI320 is colored the same as in Figure 1A. Residues Lys300 and Glu305 of helix αR2 and the interacting residues including Glu102 of the hinge region and Ser291 and Lys295 of helix αR1 are shown with ball-and-stick models and colored in cyan. The hydrogen-bonding interactions are indicated with dashed lines. **D** Comparison of helix αR2 (residues 299–314) in the CaMKI320-ATP complex (red) with the corresponding region in the CaM-CaMKI peptide complex (blue, PDB code 1MXE) [27] and the CaMKIIδ-CaM complex (gray, PDB code 2WEL) [29]. For simplicity, only CaM and the CaM-binding segment of CaMKII in the CaMKIIδ-CaM complex are shown.

Although the CaMKI320-ATP and CaMKI315-ATP complexes show substantial differences from the apo CaMKI320 in the activation segment and the nucleotide-binding site, in all three structures helix αR1 (residues 287–297) of the autoinhibitory segment interlocks with helices αD and αF, and the surrounding structure elements including the αD-αE loop and helix αE adopt essentially identical conformations (Figure 4A). Superposition of these structures with Akt/PKB-GSK3β shows that the residues at the P(-5) and P(-6) positions of the substrate peptide would collide with the C-terminal part of helix αR1, suggesting that helix αR1 might exert the inhibitory function via occlusion of part of the substrate-binding site (Figure 2D). In contrast, in the CaMKI293-ATP, the C-terminus (residues 278–293) is disordered, and consequently helix αD and the αD-αE loop show evident conformational changes. Particularly, Tyr113 of the αD-αE loop moves about 4.5 Å to insert into a narrow space surrounded by helices αD, αE, and αF, and its side chain is re-oriented to form hydrophobic interactions with Leu103 and Ile107 of helix αD, Leu121 of helix αE, and Leu211 of αF, stabilizing helix αD in the new conformation and preventing the re-binding of the autoinhibitory segment (Figure 4B). Concurrently, the space originally occupied by the C-terminal part of helix αR1 is vacant for substrate binding. The conformations of helix αD and the αD-αE loop particularly Tyr113 in CaMKI293-ATP are similar to those of the equivalents in Akt/PKB-GSK3β. In addition, Tyr113 is strictly conserved across the CaMK family (Figure 1B), underscoring the importance of this residue and supporting our notion that the conformational changes of helix αD and the αD-αE loop are critical for CaMKI activation. It was reported that mutation of Ile294 and Phe298 to Ala resulted in a modest level of CaM-independent activity, and additional mutation of Ile286 and Val290 to Ala resulted in significant increase of the basal activity [15]. It is likely that mutation of these hydrophobic residues of the autoinhibitory segment might dislodge helix αR1 from the substrate-binding site, leading to conformational changes of helix αD and the αD-αE loop and hence partial activation of the kinase.

It was also reported that CaMKI297 is constitutively active albeit with a relatively low activity [15]. CaMKI297 contains all the residues that form helix αR1 in the apo CaMKI320 and the CaMKI320-ATP and CaMKI315-ATP complexes but its activity is not completely inhibited, suggesting that the CaM-binding segment might play some role in facilitating the autoinhibitory segment in the inhibition of the activity. In the rat CaMKI320, the CaM-binding segment forms a long loop that curves into the entry of the ATP-binding site followed by a short αR2 helix that interacts with the N lobe of the kinase [16] (Figure 2A). Particularly, Lys300 of the αR1-αR2 loop forms a salt bridge with the strictly conserved Glu102 of the hinge region, which might prohibit Glu102 from binding ATP or the substrate [16]. Intriguingly, in the CaMKI320-ATP complex, the CaM-binding segment mainly forms a long αR2 helix which protrudes away from the catalytic core (Figure 2A). A detailed analysis indicates that helix αR2 of this conformation plays an important role in the maintenance of an inactive state of the enzyme through interaction with Glu102 and stabilization of the inactive conformations of helices αR1 and αD. Specifically, Lys300 on helix αR2 also forms a salt bridge with Glu102; this interaction does not abrogate the ability of Glu102 to bind ATP as Glu102 still makes hydrogen-binding interactions with the 2′- and 3′-hydroxyls of the ribose moiety of ATP (Figure 4C), however, it could have an impact on its ability to bind the substrate as Glu102 is also suggested to play a role in the recognition and binding of Arg at P(-3) of the substrate (see discussion above) (Figure 2D). In addition, the N-terminal part of helix αR2 would have steric conflicts with the C-terminal part of helix αD in the CaMKI293-ATP complex, preventing helix αD from adopting an active conformation. Furthermore, the side chain of Gln305 of αR2 forms two hydrogen-bonding interactions with the side chains of Ser291 and Lys295, and thus helix αR2 also contributes to stabilization of helix αR1 in the inactive conformation (Figure 4C).

To better understand how CaM binds to and activated CaMKI, we superposed the available structures of kinases with the CaM-binding and/or autoinhibitory segments including other CaMK members and the death-associated protein kinase (Figure 4D and S2A). In the crystal structure of CaM in complex with a peptide corresponding to the CaM-binding segment of CaMKI (residues 294–318), the peptide forms a long α-helix [27]. The NMR spectra of CaM bound to either CaMKI320 or a similar peptide (residues 299–320) were virtually identical [28], indicating that the binding mode observed in the CaM-CaMKI peptide complex might be retained in the binding of CaM with CaMKI. Superposition of the CaMKI320-ATP structure with the CaM-CaMKI peptide structure [27] and the recently reported CaMKIIδ-CaM structure [29] based on the CaM-binding segment (corresponding to residues 299–314 of CaMKI) demonstrates that CaM binds to CaMKI and CaMKII in a similar mode, and helix αR2 in CaMKI320-ATP encompasses almost all the residues required for direct interaction with CaM (Figure 4D). Therefore, the position and conformation of the CaM-binding segment in CaMKI320-ATP correspond to a biologically relevant state of CaMKI ready for CaM binding. On the other hand, a short region at the N-terminus of CaM appears to have steric conflicts with helix αD in the CaMKI320-ATP complex, indicating that proper conformational change or dissociation of the N-terminal part of helix αR2 and the autoinhibitory segment is required prior to the binding of the complete αR2 helix by CaM (Figure S2B).

In the structure of CaMKIV in complex with an inhibitor (PDB code 2W4O), the residues corresponding to helices αR1 and αR2 form a unified long α-helix which folds along the catalytic core to interact with both N lobe and C lobe. Superposition of residues 299–314 (helix αR2) of the CaMKI peptide in the CaM-CaMKI peptide structure with the corresponding region (residues 322–337) in the CaMKIV structure shows severe steric conflicts between CaM and the N lobe of CaMKIV (Figure S2C), indicating that the regulatory region with such a long helical conformation is unsuitable for CaM binding. Given the conservation of the regulatory region across the CaMK family (Figure 1B), the CaM-binding segment in CaMKIV is likely to assume a conformation as in CaMKI320-ATP prior to CaM binding. The regulatory scheme of the CaMKs by CaM seems to be different from that of death-associated protein kinase, in which the autoregulatory domain forms a long helix and the bound CaM takes an extended conformation [30].

### Relay of conformational changes upon activation by CaM binding

Our structural data suggest that the binding of helix αR2 by CaM would require conformational change or dissociation of the autoinhibitory segment from the catalytic core. Consistently, structural studies of CaMKII show that with respect to the autoinhibited kinase the binding of CaM to CaMKIIδ leads to dissociation of the autoinhibitory and CaM-binding segments from the catalytic core, resulting in the rearrangement of helix αD [29]. Given that the conformation of helix αD in the active CaMKI293-ATP complex is essentially the same as that in the CaMKIIδ-CaM complex [29] and the Akt/PKB-GSK3β complex [23], these studies together support the notion that the binding of CaM to CaMKs leads to dissociation of the regulatory region from the catalytic core and the consequent rearrangement of helix αD into an active conformation, which is critical for the activation of CaMKs.

**Figure 5:**
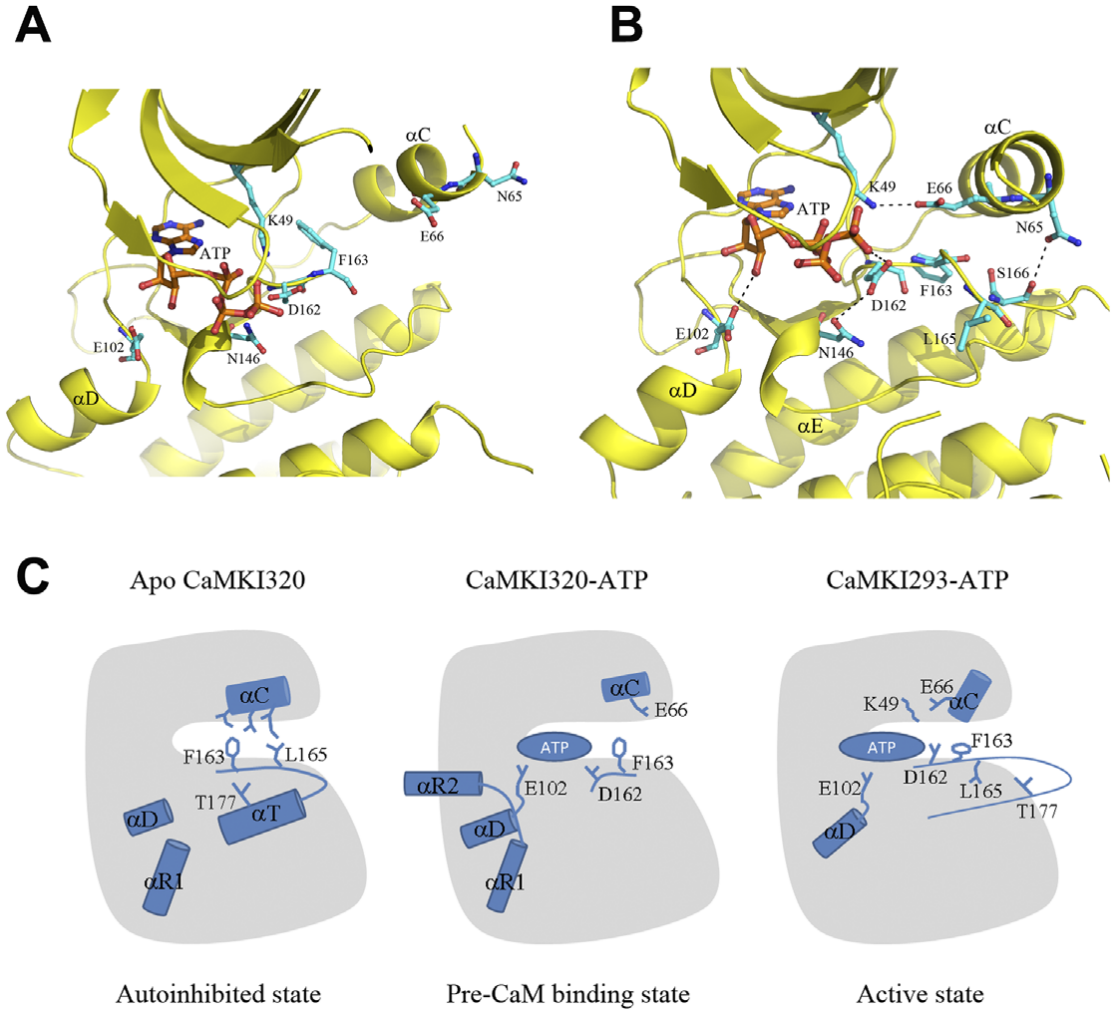
Model of the regulation of CaMKI. **A** Structure of the catalytic site in the CaMKI320-ATP complex. **B** Structure of the catalytic site in the CaMKI293-ATP complex. Comparison of these two structures reveals the conformational changes upon activation. CaMKI is colored in yellow and the bound ATP in orange. In **B**, the residues that play important roles in the conformational changes are shown with ball-and-stick models in cyan. **C** A schematic diagram showing the proposed model of the regulation of CaMKI. The conversion of CaMKI from an autoinhibited state to an inactive, pre-CaM binding state, and subsequently an active state is illustrated. The α-helices that undergo conformational changes during the conversion and the key residues are indicated with cylinders and stick models, respectively. ATP is represented with an oval.

Now the question is how the activation of the kinase takes place after the removal of the restraint of the regulatory region on helix αD. Comparisons of the apo CaMKI320, CaMKI320-ATP, and CaMKI293-ATP structures provide some clues. In CaMKI320-ATP, helix αD takes an inactive conformation, and the side chain of Glu102 of the hinge region forms hydrogen-binding interactions with both 2′- and 3′-hydroxyls of the ribose moiety of ATP, pulling the ribose and phosphate groups deep into the nucleotide-binding site (Figure 5A). In CaMKI293-ATP, with the rearrangement of helix αR1 and the consequent relaxation of helix αD, the constraint on Glu102 by Lys300 of helix αR2 is removed. The side chain of Glu102 displays a positional displacement of 1.5 Å towards the catalytic site and binds to the 3′-hydroxyl rather than both hydroxyls of the ribose of ATP (Figure 5B). Concomitantly, ATP shifts upwards by about 1.5 Å, and several residues at the N-terminal part of the activation segment show notable conformational changes and form different interactions. Specifically, Asp162 interacts with the γ-phosphate group of ATP, and the side chain of the following Phe163 rotates about 30° towards helix αE, leaving space for the rotation of helix αC towards strand β3. In addition, the side chain of Leu165, which is disordered in CaMKI320-ATP and CaMK315-ATP, becomes evident and takes a position opposite to that in the apo CaMKI320 (Figures 2C, 5A and 5B), and therefore no longer participates in the interactions between the activation segment and helix αC. Accompanying these changes, several structure elements of the N lobe, in particular strand β3 and helix αC, also undergo substantial conformational changes. Lys49 of strand β3 moves upwards along with ATP by 2.5 Å and forms a salt bridge with Glu66 of helix αC. Concurrently, the side chain of Asn65 on helix αC is hydrogen-bonded to the side chain of Ser166 on the activation segment, stabilizing the N-terminal part of the activation segment. With the salt bridge between Lys49 and Glu66 established and helix αC rotated to the active position, the catalytic site is assembled.

## Discussion

### Model of autoinhibition and activation of CaMKI

The structures of CaMKI presented in this study represent three different conformational states of the kinase. Analyses of these structures reveal conformational differences particularly in the regulatory region, the activation segment, and the nucleotide-binding site. The structural data are in consistency with the previous biochemical and structural data including the unresponsiveness of the inactive CaMKI to CaMKK [26], the constitutive albeit relatively low activity of CaMKI297 [15], and the adoption of a helical conformation of the CaM-binding segment for CaM binding [27]. Based on these results, we can propose a model of the regulation mechanism of CaMKI (Figure 5C). In the apo form, CaMKI exists in an autoinhibited state which is represented by the apo CaMKI320 structure (Figure 5C, autoinhibited state). In this state, the autoinhibitory segment (helix αR1) together with the CaM-binding segment (helix αR2) places strong constraints on helix αD of the C lobe to stabilize it in an inactive conformation, which blocks the binding of the substrate residues N-terminal to the P0 position. Meanwhile, the activation segment assumes a loop conformation at the N-terminus and a helical conformation at the C-terminus (helix αT). This unique conformation of the activation segment also contributes to the inhibition of CaMKI in three possible ways: (1) it interacts with the hydrophobic surface of helix αC via the highly conserved Phe163 and Leu165 to restrain helix αC in an inactive position; (2) it sequesters Thr177 and hence prevents its phosphorylation by CaMKK; (3) it prevents the binding of the substrate residues C-terminal to the P0 position. The apo CaMKI in the autoinhibited state is amenable to nucleotide binding. ATP binding can induce conformational changes at the nucleotide-binding site and the activation segment, leading to the formation of an inactive, pre-CaM binding state which is represented by the CaMKI320-ATP and CaMKI315-ATP structures (Figure 5C, pre-CaM binding state). In this state, the activation segment is largely disordered and its restraint on helix αC is alleviated. Nevertheless, helix αC remains in an inactive conformation and the catalytic site is only partially assembled. On the other hand, the CaM-binding segment adopts a long helical conformation that is ready for CaM binding. Upon CaM binding with the CaM-binding segment, the regulatory region is dissociated from the catalytic core, leading to the formation of an active state which is represented by the CaMKI293-ATP structure (Figure 5C, active state). The dissociation of the regulatory region releases the constraints on helix αD, resulting in rearrangements of helix αD, the preceding β5-αD loop (hinge region), and the following αD-αE loop. These conformational changes in the C lobe are propagated in long range to the N lobe via Glu102 of the hinge region, the bound ATP, and Asp162, Phe163, and Leu165 of the activation segment, which leads to the formation of the salt-bridging interaction between Lys49 of strand β3 and Glu66 of helix αC and hence completes the assembly of the catalytic site. In summary, the regulatory region, the activation segment, and the nucleotide-binding site of CaMKI are correlated together, and upon the binding of ATP and CaM these regions interplay in a concerted way to regulate the activity states of the kinase.

### Comparison with helical conformations of the activation segment in other kinases

As written by Noble *et al.* in paraphrase of the opening sentence of Anna Karenina, “all active kinases are alike but an inactive kinase is inactive after its own fashion” [31]. The CaMKI structures presented here unexpectedly reveal a rare helical conformation of the activation segment. Among >5000 kinase structures in Protein Data Bank, there are only very few in which the activation segment contains an α-helix, including serine/threonine kinases CDKs [32], [33] and Nek2 [34], [35] and tyrosine kinases Src/Hck (reviewed in [36]) and EGFR [37], [38]. In most of these structures, the N-terminal part of the activation segment folds into a short α-helix which interacts with helix αC to constrain it in an inactive position [32], [34], [36], [37], [38]. Hydrophilic interactions of this part with helix αC, particularly with an invariant Glu, have been suggested to be critical for maintenance of the inactive conformation of the kinase [32]. In addition, hydrophobic interactions between this part and helix αC have also been demonstrated to be important. For example, in the inactive EGFR, a Leu immediately following the DFG motif (Leu834) and an adjacent Leu (Leu837) on the short α-helix of the activation segment pack against the hydrophobic side of helix αC to stabilize its inactive conformation [37], [38]. Mutations of these two residues to polar ones (L834R and L837E) have been found in lung cancer patients, and the EGFR kinase domain carrying the L834R mutation displays a substantially increased activity, underscoring the importance of the two residues in the inhibition of the kinase activity [37].

In the CaMK family, only the Leu residue equivalent to Leu834 of EGFR is conserved, corresponding to Leu165 of CaMKI. In the apo CaMKI320, although the N-terminal part of the activation segment takes a loop conformation rather than a helical conformation, the side chain of Leu165 is also oriented towards helix αC and forms hydrophobic interactions with residues of helix αC, contributing to the stabilization of helix αC in the inactive conformation. The specific conformation of Leu165 is apparently associated with the αT-containing activation segment, as in the rat CaMKI320 and CaMKI320-ATP and CaMKI315-ATP structures, Leu165 is disordered, while in the CaMK293-ATP structure, the side chain of Leu165 points to an opposite direction towards the catalytic site.

In some Nek2 structures, the C-terminal part of the activation segment adopts a helical conformation and occupies a position similar to that of helix αT in CaMKI320, including the wild-type Nek2 in complex with ADP, and the T175A mutant Nek2 in apo form and in complex with an ATP analog [35], indicating that the helical conformation is formed independent of Thr175 and nucleotide binding. Consistently, in the wild-type Nek2, Thr175 (equivalent to Thr177 of CaMKI) is exposed to the solvent; whereas in CaMKI320, Thr177 points to the catalytic site to interact with Tyr195 and hence is inaccessible to CaMKK. Considering the previous report that Thr177 of the full-length CaMKI cannot be phosphorylated in the absence of CaM [26], the helical conformation of the activation segment that is characterized by the sequestration of Thr177 is likely to be adopted by the autoinhibited full-length CaMKI.

Drugs that specifically target a specific inactive conformation of kinases can achieve high selectivity, which has been exemplified by Gleevec [31], [39]. Therefore, it is noteworthy that the newly identified helical conformation of the activation segment in CaMKI combined with the differences between CaMKI and other kinases in this region could be exploited in the development of inhibitors with high selectivity that can specifically lock CaMKI in this unique inactive conformation.

### Accession Codes

The crystal structures of the apo CaMKI320, the CaMKI320-ATP complex, the CaMKI315-ATP complex, and the CaMKI293-ATP complex have been deposited with the RCSB Protein Data Bank under accession codes 4FG7, 4FG8, 4FG9, and 4FGB, respectively.

## Supporting Information

Figure S1 **Analysis of the interface between two two-fold symmetry-related molecules in the CaMKI structures.** **A** The interface in the apo CaMKI320 structure. The interface is formed mainly between helix αT of one molecule (in green) and helices αG and αEF of the other (in cyan, denoted with apostrophes), and is stabilized mainly by hydrophobic interactions. The involved residues in the hydrophobic interactions are shown with ball-and-stick models and colored accordingly. **B** The interface in the CaMKI320-ATP structure. The interface is comparable to that in the apo CaMKI320 structure, and helices αG and αEF occupy similar positions as those in the apo CaMKI320 structure; however, helix αT is largely disordered in one molecule and completely disordered in the other.
(TIF)

Figure S2 **Regulatory segments of CaMKs.** **A** Comparison of the overall structures of kinases with the CaM-binding and/or autoinhibitory segments including human CaMKIα (the CaMKI315-ATP structure determined in this study, green), rat CaMKIα (PDB code 1A06, cyan), human CaMK1δ (2JC6, magenta), human CaMK1γ (2JAM, yellow), human CaMK2α (2VZ6, wheat), human CaMK2β (3BHH, grey), human CaMK2δ (2VN9, sky-blue), human CaMK2γ (2V7O, orange), human CaMKIV (2W4O, blue), and human death-associated kinase 1 (2X0G, red). **B** Docking of CaM to the CaMKI320-ATP complex based on the CaM-binding segment (helix αR2, residues 299–314). CaM is shown with a ribbon-and-surface representation in gray. CaMKI320 is shown with a ribbon representation in magenta. **C** Superposition of the structure of the CaM-CaMKI peptide complex and the structure of CaMKIV in complex with an inhibitor (blue, PDB code 2W4O) based on the CaM-binding segment (residues 299–314 of CaMKI and residues 322–337 of CaMKIV) shows severe steric conflicts between CaM and the N lobe of CaMKIV.
(TIF)
