# Supplementary figures and images for: Crystal Structures of Human CaMKIα Reveal Insights into the Regulation Mechanism of CaMKI
Source: PLoS One. 2012 Sep 20;7(9):e44828. doi: 10.1371/journal.pone.0044828 (PMC3447817; doi:10.1371/journal.pone.0044828)

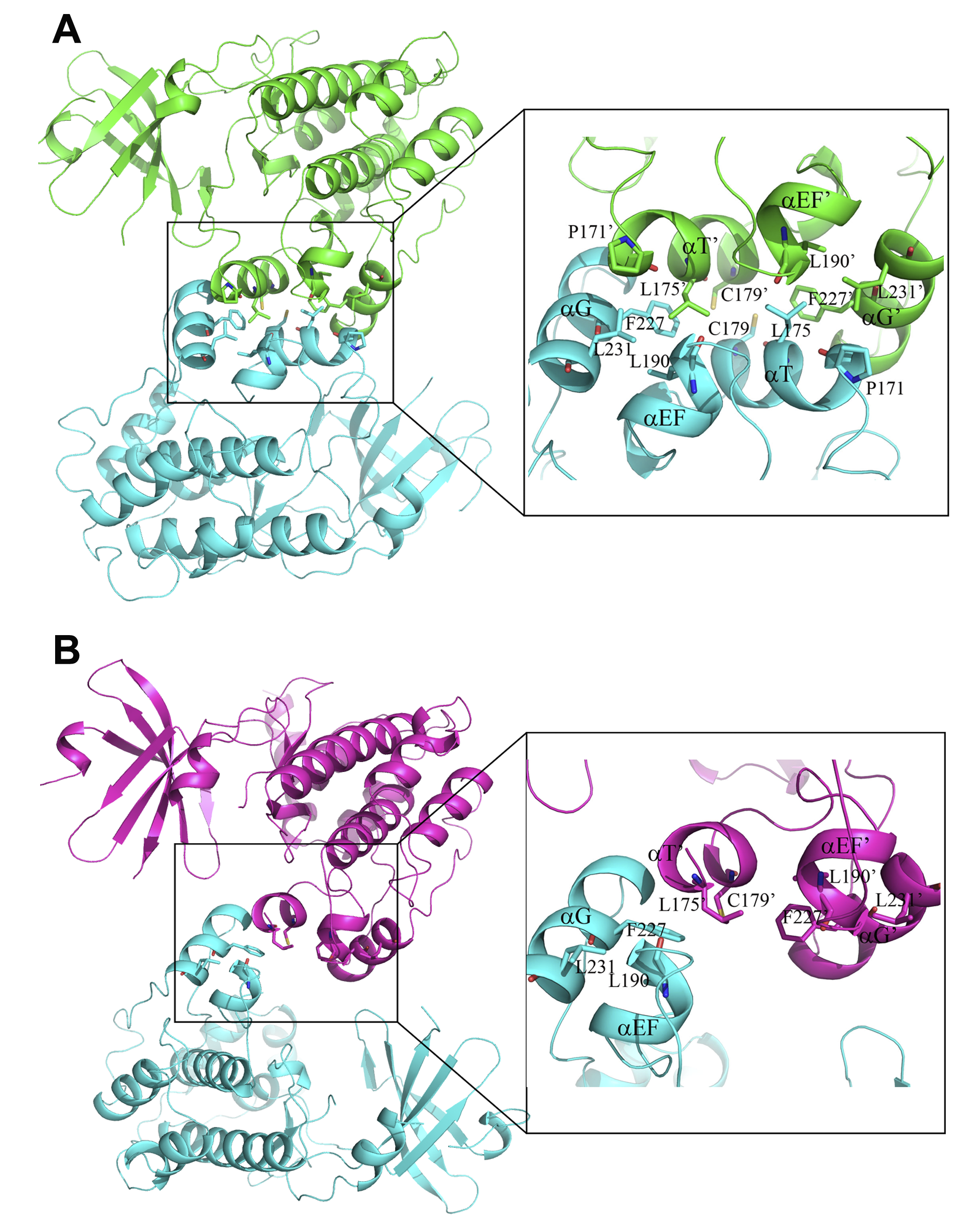

Supplement: Figure S1 — Analysis of the interface between two two-fold symmetry-related molecules in the CaMKI structures. A The interface in the apo CaMKI320 structure. The interface is formed mainly between helix αT of one molecule (in green) and helices αG and αEF of the other (in cyan, denoted with apostrophes), and is stabilized mainly by hydrophobic interactions. The involved residues in the hydrophobic interactions are shown with ball-and-stick models and colored accordingly. B The interface in the CaMKI320-ATP structure. The interface is comparable to that in the apo CaMKI320 structure, and helices αG and αEF occupy similar positions as those in the apo CaMKI320 structure; however, helix αT is largely disordered in one molecule and completely disordered in the other. (TIF) [file pone.0044828.s001.tif]

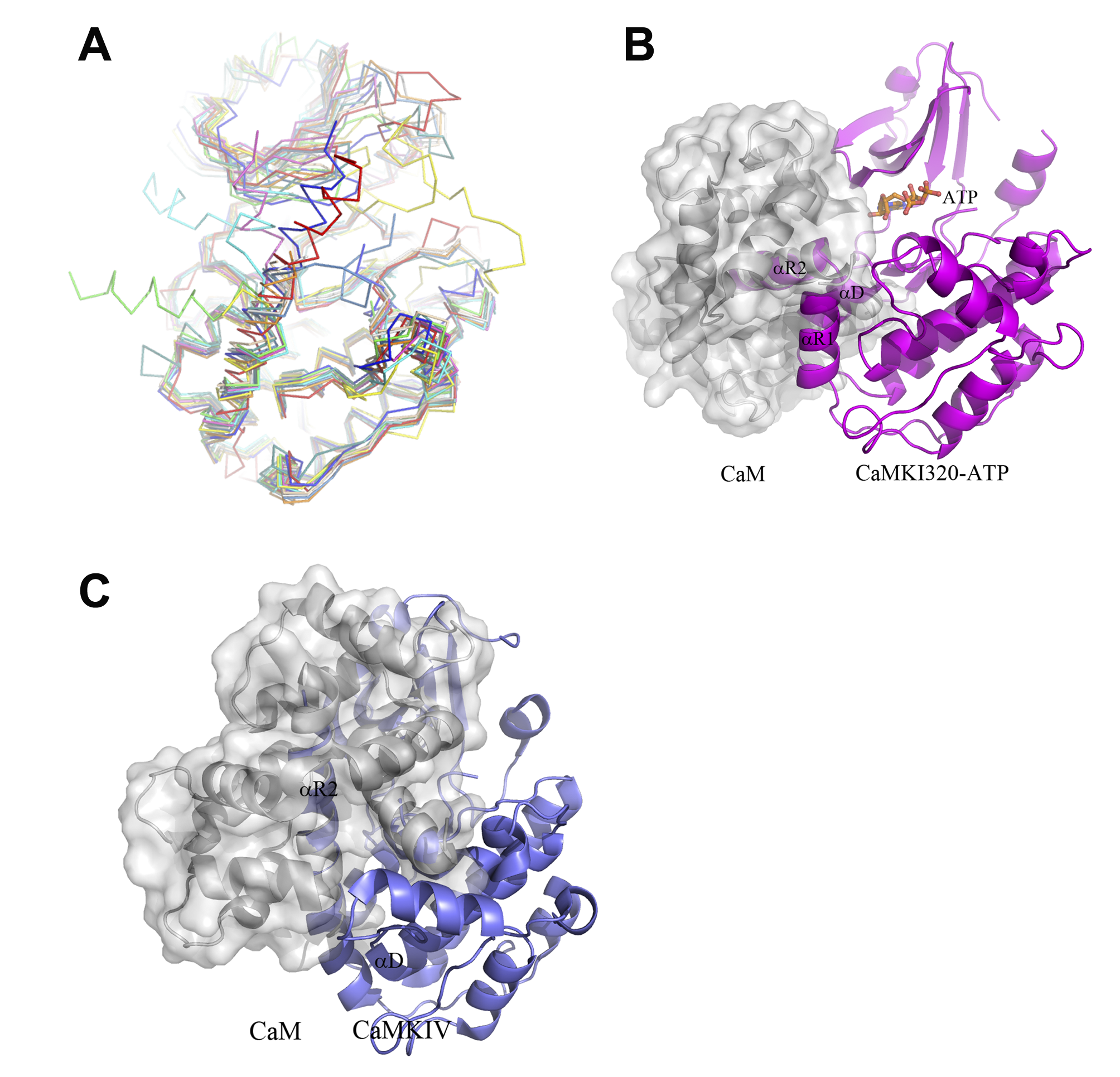

Supplement: Figure S2 — Regulatory segments of CaMKs. A Comparison of the overall structures of kinases with the CaM-binding and/or autoinhibitory segments including human CaMKIα (the CaMKI315-ATP structure determined in this study, green), rat CaMKIα (PDB code 1A06, cyan), human CaMK1δ (2JC6, magenta), human CaMK1γ (2JAM, yellow), human CaMK2α (2VZ6, wheat), human CaMK2β (3BHH, grey), human CaMK2δ (2VN9, sky-blue), human CaMK2γ (2V7O, orange), human CaMKIV (2W4O, blue), and human death-associated kinase 1 (2X0G, red). B Docking of CaM to the CaMKI320-ATP complex based on the CaM-binding segment (helix αR2, residues 299–314). CaM is shown with a ribbon-and-surface representation in gray. CaMKI320 is shown with a ribbon representation in magenta. C Superposition of the structure of the CaM-CaMKI peptide complex and the structure of CaMKIV in complex with an inhibitor (blue, PDB code 2W4O) based on the CaM-binding segment (residues 299–314 of CaMKI and residues 322–337 of CaMKIV) shows severe steric conflicts between CaM and the N lobe of CaMKIV. (TIF) [file pone.0044828.s002.tif]
